# Incorporating information of causal variants in genomic prediction using GBLUP or machine learning models in a simulated livestock population

**DOI:** 10.1186/s40104-025-01250-5

**Published:** 2025-08-19

**Authors:** Jifan Yang, Mario P. L. Calus, Yvonne C. J. Wientjes, Theo H. E. Meuwissen, Pascal Duenk

**Affiliations:** 1 Animal Breeding and Genomics, Wageningen University & Research, Wageningen, 6700 AH The Netherlands; 2 Faculty of Life Sciences, Norwegian University of Life Sciences, Ås, 1432 Norway

**Keywords:** GBLUP, Genomic prediction, Machine learning, QTL, Random forest, Support vector regression

## Abstract

**Background:**

Genomic prediction has revolutionized animal breeding, with GBLUP being the most widely used prediction model. In theory, the accuracy of genomic prediction could be improved by incorporating information from QTL. This strategy could be especially beneficial for machine learning models that are able to distinguish informative from uninformative features. The objective of this study was to assess the benefit of incorporating QTL genotypes in GBLUP and machine learning models. This study simulated a selected livestock population where QTL and their effects were known. We used four genomic prediction models, GBLUP, (weighted) 2GBLUP, random forest (RF), and support vector regression (SVR) to predict breeding values of young animals, and considered different scenarios that varied in the proportion of genetic variance explained by the included QTL.

**Results:**

2GBLUP resulted in the highest accuracy. Its accuracy increased when the included QTL explained up to 80% of the genetic variance, after which the accuracy dropped. With a weighted 2GBLUP model, the accuracy always increased when more QTL were included. Prediction accuracy of GBLUP was consistently higher than SVR, and the accuracy for both models slightly increased with more QTL information included. The RF model resulted in the lowest prediction accuracy, and did not improve by including QTL information.

**Conclusions:**

Our results show that incorporating QTL information in GBLUP and SVR can improve prediction accuracy, but the extent of improvement varies across models. RF had a much lower prediction accuracy than the other models and did not show improvements when QTL information was added. Two possible reasons for this result are that the data structure in our data does not allow RF to fully realize its potential and that RF is not designed well for this particular prediction problem. Our study highlighted the importance of selecting appropriate models for genomic prediction and underscored the potential limitations of machine learning models when applied to genomic prediction in livestock.

**Supplementary Information:**

The online version contains supplementary material available at 10.1186/s40104-025-01250-5.

## Background

The extensive use of genomic prediction (GP) has significantly increased the genetic improvement in the animal breeding industry [1, 2]. In GP, causal variants, i.e., quantitative trait loci (QTL) that influence the trait of interest, are assumed to be in linkage disequilibrium (LD) with at least one single nucleotide polymorphism (SNP) marker. When enough markers are used, the markers are expected to explain together the genetic variance for the trait and, therefore, can be used to predict breeding values for the selection candidates without knowing the actual causal variants [3].

Common models used to predict genomic breeding values are GBLUP and ssGBLUP which assume that all markers explain the same amount of genetic variance [2, 4, 5]. This assumption can be relaxed by using a method named weighted GBLUP which can put more weight on markers that are known to explain a larger proportion of the genetic variance [6]. The assumption of equal variance for all markers can also be relaxed by using Bayesian models. These models require parameters that determine the distribution of marker effects and these parameters can be assumed known or can be estimated from the data [3]. For example, BayesCπ assumes that only a small proportion of markers have an effect and estimates the proportion of markers with no effect (i.e., *π*) in the model [7].

Another option for GP is using machine learning (ML) methods, which do not make assumptions about the distribution of marker effects and the underlying parameters. Examples of ML models applied to genomic prediction are Bayesian neural network [8, 9], reproducing kernel Hilbert spaces regression [10, 11], support vector regression (SVR) with non-linear kernels [12], and decision tree based algorithms such as random forest (RF) [13, 14]. In some of these studies, machine learning models yielded higher prediction accuracy than GBLUP [8, 15, 16], while others did not find benefits of ML over GBLUP [17–20].

Studies aiming to improve accuracy by increasing the number of markers have generally failed. In cattle, there was generally no improvement in accuracy when using more than 100k markers compared to 50k markers, which may be because only relatively few of all whole genome sequence (WGS) variants add useful additional information, while all other added variants may only dilute the signals captured by the marker loci [21–23]. To reduce the impact of added noise when using more markers, feature selection was introduced in some Bayesian models. So far, applications of such models to imputed WGS data did not yield improvements in accuracy compared to using a high density marker panel, which may be explained by the structure of the training dataset [22, 24]. Alternatively, ML models can do feature selection by assigning different weights to genetic variants. Two studies reported that a smaller number of markers resulted in a higher prediction accuracy [19, 20]. Because the inclusion of an excessive number of markers can negatively impact ML performance due to the risk of overfitting. However, as far as we know, it is not yet known whether the inclusion of informative genetic variants such as information on causal loci in ML models can improve the prediction accuracy.

As an alternative to feature selection, some studies have attempted to take a two-step approach. First, a pre-selection of markers was made based on results from genome-wide association studies (GWAS). Second, those pre-selected markers were included as a separate genetic effect in a GBLUP model. Most of these studies have reported that this strategy improved the prediction accuracy [25–30]. The reason for this improvement is that the separation of pre-selected markers prevents the dilution of the genetic signals among the markers that are in weak LD with the causal variants. These studies with the two step approach only focussed on a limited number of top-SNPs. However, due to recent developments in quantitative genetics and molecular genomics, combined with advances in cost-effective sequencing technologies, we can expect that more and more causal loci will be identified [31]. However, it is still unclear whether it is optimal to include all preselected markers, or only the ones that explain most of the genetic variance.

Machine learning models differ from GBLUP because they are not specifically designed to predict breeding values. Instead, ML models predict the provided input, such as phenotypes. This fundamental difference might lead to ML models being less accurate than GBLUP for predicting breeding values. However, both approaches can simply be viewed as prediction models that use phenotypes as input, and genotypes as features. Furthermore, some simulation studies have shown that ML models can outperform GBLUP in terms of the correlation between predicted values and breeding value [8, 32]. It is therefore interesting to compare the accuracy between ML models and GBLUP, eventhough they aim to predict different things.

The objective of this study was to assess the benefit of incorporating QTL information in GBLUP and machine learning models for genomic prediction accuracy, and to find the proportion of genetic variance explained by QTL included in the model at which the highest prediction accuracy is achieved. Our approach was based on simulations of a livestock population in which all the QTL genotypes and effects were known. We used GBLUP, random forest and support vector regression for analysis. The two machine learning methods are representative of two categories of methods, namely ensemble methods (RF) and kernel methods (SVR). In addition, we investigated the reason why there is a difference in prediction accuracy between different models.

## Methods

### Simulation

The data was simulated with QMSim [33], using the parameter file in Additional file 1. The simulation process tried to mimic a historic dairy cattle population. It is good to note that our aim was not to completely resemble a dairy cattle population, but to simulate a population under selection with a comparable genome structure as livestock species to test the different genomic prediction models. In the first generation of the historic population, there were 2,800 individuals with equal number of both sexes. The population size gradually decreased to 2,000 in generation 2,250, and further gradually decreased to 200 in generation 2,500 to generate linkage disequilibrium. After that, the population size linearly increased to 10,000 in generation 2,505. After the historic population, 20 generations (numbered as 1–20) of 5,000 animals with an equal sex ratio and litter size of 1 were simulated, using 20 males and 5,000 females as parents in each generation. In each new generation, all male parents and half of the female parents were replaced. For both males and females, new parents were selected using pedigree-based estimated breeding values for a polygenic selection trait with an accuracy of 0.8.

We simulated 29 pairs of autosomes that each had a size of 100 cM. On each autosome, 350 quantitative trait loci (QTL) and 2,000 SNP markers were randomly placed. Together, the QTL explained all the genetic variance (*h*^2^ = 0.25) of the trait under selection, and the QTL effects were sampled from a gamma distribution with a shape parameter of 0.42. True breeding values (TBV) were calculated by multiplying the genotypes (coded as allele counts: 0, 1, 2) with the effects of the counted QTL allele, and phenotypes were calculated as the sum of TBV and a random environmental effect.

The final dataset contained the genotypes of animals from generation 0 to 20, and phenotypes of the selected trait from generation 0 to 15, which were available for both sexes. For generation 11–15, 2,500 animals in each generation were randomly chosen to be included in the training dataset. For generation 16, 500 animals were randomly chosen to be included in the test set. Therefore, there are 12,500 animals in the training dataset and 500 animals in the test dataset. We simulated 20 replicates in total.

**Fig. 1:**
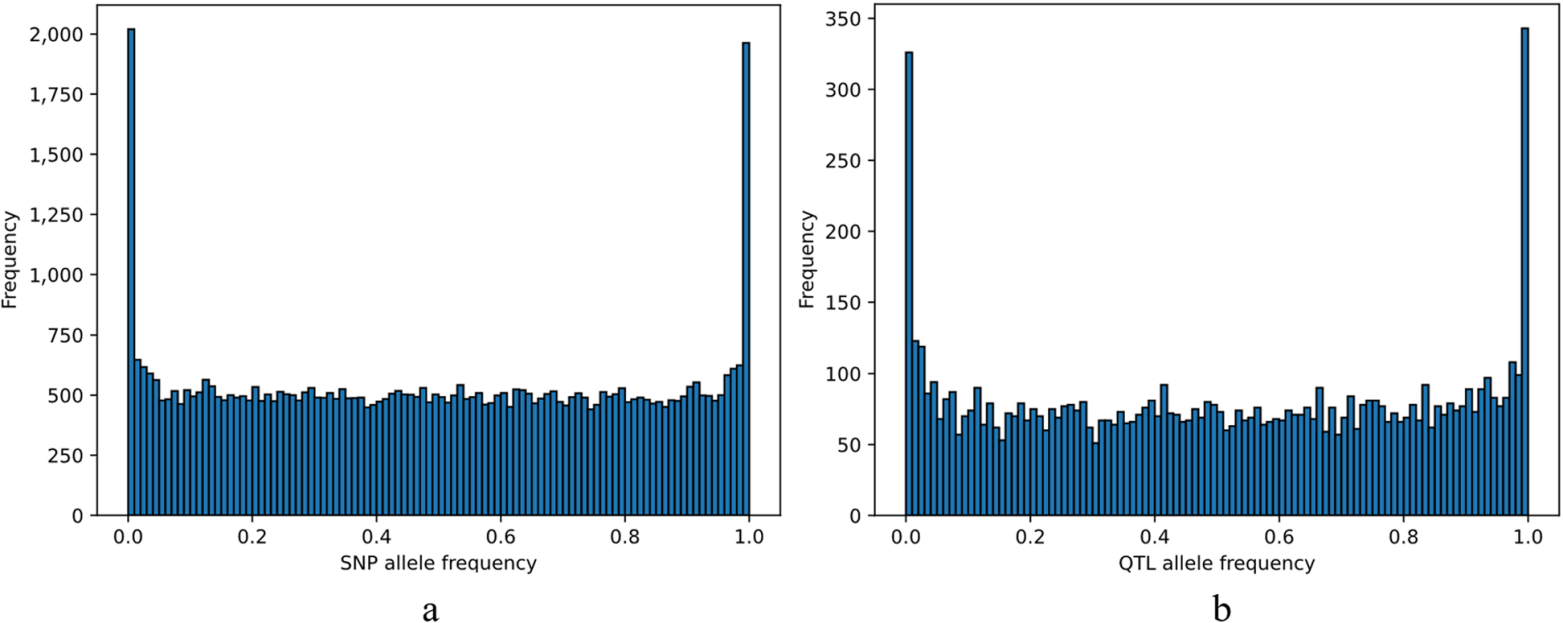
Allele frequency distribution of segregating SNPs (**a**) and QTL (**b**) in one replicate. Note that SNPs/QTL that were fixed for a single allele (i.e., with allele frequency of 0 or 1) are not represented in this graph

**Fig. 2:**
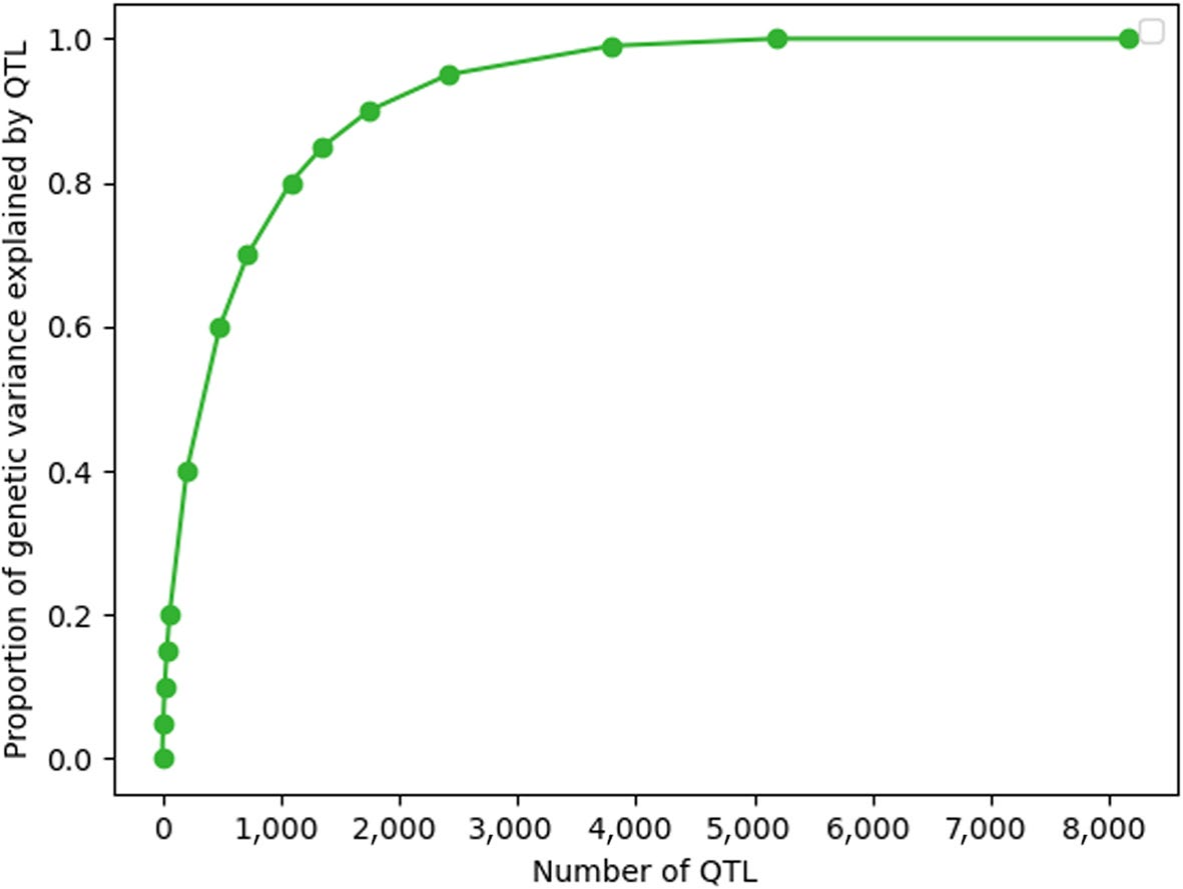
The cumulative proportion of genetic variance explained by QTL versus the number of QTL averaged across replicates. The QTL are ordered from QTL that explain the most genetic variance to QTL that explain the least genetic variance

After the simulation, the distribution of allele frequencies of the segregating SNPs and QTL were U-shaped (Fig. 1). The average total number of segregating SNPs among 20 replicates was 55,043.2 ± 82.9 and there were on average 8,169.8 ± 44.0 segregating QTL in generation 0. We sorted the QTL based on the amount of variance that they explained. So, with increasing number of QTL, the genetic variance explained by QTL increased non-linearly (Fig. 2). On average, the 8.6 ± 1.4 most important QTL together explained 5% of the genetic variance, and the 3,789.5 ± 78.6 most important QTL explained 99% of the genetic variance, while the 1,392.0 ± 48.1 least important QTL explained only 1% of the genetic variance. 2,988.4 ± 116.9 QTL cumulatively explained less than 0.0005% of the genetic variance, and we excluded these QTL.

### Estimating breeding values

#### GBLUP

The first model used to estimate breeding values was GBLUP. With the GBLUP model, we included one random genomic effect without separating QTL from SNP markers. The used model was

$${\varvec{y}}={\bf 1}{\mu}+{\varvec{Z}}{\varvec{u}}+{\varvec{e}},$$

where ${\varvec{y}}$ is the vector of phenotypes, *µ* is the overall mean, $\bf 1$ is a vector of ones, ${\varvec{u}}$ is the vector of genomic breeding values of SNP and QTL following a normal distribution *N*(**0**, ${\varvec{G}}{\sigma }_{u}^{2}$), ${\varvec{Z}}$ is an incidence matrix that links ${\varvec{u}}$ to ${\varvec{y}}$, and ${\varvec{e}}$ is the vector of random errors, following a normal distribution of *N*($0$, ${\varvec{I}}{\sigma }_{e}^{2}$), where ${\sigma }_{e}^{2}$ is the residual variance. ${\varvec{G}}$ is the genomic relationship matrix which was constructed using genotypes of all SNP and QTL markers, and method (1) of VanRaden [34]:

$${\varvec{G}}=\frac{\varvec{WW}^{\prime}}{2{\sum }_{j}{p}_{j}(1-{p}_{j})}\boldsymbol{ },$$

where ${\varvec{W}}$ includes the centered allele counts of the genotypes, with elements *w*_*ij*_ = *x*_*ij*_* − 2p*_*j*_, where *x*_*ij*_ is an element of matrix ${\varvec{X}}$ containing the genotype for individual *i* at locus *j* coded as 0, 1, 2; and *p*_*j*_ is the frequency of the allele for which the homozygous genotype at locus* j* is coded as 2.

#### 2GBLUP

In the 2GBLUP model, we separated the pre-selected QTL and SNP markers by including two random genomic effects in the model allowing for different proportions of the genetic variance being explained by QTL and SNPs:

$${\varvec{y}}={\bf 1}{\mu} +{\varvec{Z}}{{\varvec{u}}}_{1}+{\varvec{Z}}{{\varvec{u}}}_{2}+{\varvec{e}},$$

where ${\varvec{y}}$, $\bf 1$, *µ* and ${\varvec{e}}$ are the same as for GBLUP, while ${{\varvec{u}}}_{1}$ and ${{\varvec{u}}}_{2}$ are vectors of genomic breeding values based on QTL and SNP markers, respectively, which follow a normal distribution with *N*(**0**, ${{\varvec{G}}}_{1}{\sigma }_{{u}_{1}}^{2}$) and *N*(**0**, ${\varvec{G}}_{2}{\sigma }_{{u}_{2}}^{2}$), where ${\sigma }_{{u}_{1}}^{2}$ + ${\sigma }_{{u}_{2}}^{2}$ equal the additive genetic variance (i.e., ${\sigma }_{u}^{2}$ in GBLUP). ${\varvec{Z}}$ is an incidence matrix that links ${{\varvec{u}}}_{1}$ and ${{\varvec{u}}}_{2}$ to ${\varvec{y}}$. ${{\varvec{G}}}_{2}$ was constructed using SNP genotypes, and ${{\varvec{G}}}_{1}$ was constructed using QTL genotypes, using the same method as for ${\varvec{G}}$.

The underlying assumption in the 2GBLUP model when constructing ${{\varvec{G}}}_{1}$ was that all QTL explain the same amount of genetic variance, which is not true (Fig. 2). To investigate the impact of this assumption, we included a model weighted 2GBLUP (w2GBLUP), where the ${{\varvec{G}}}_{2}$ matrix was computed as before while QTL variances were used as weights to construct a weighted ${{\varvec{G}}}_{1}$ [6]:

$${{\varvec{G}}}_{1}=\frac{\varvec{WDW}^{\prime}}{2{\sum }_{j}{p}_{j}(1-{p}_{j})}\boldsymbol{ },$$

where $\varvec{D}$ is a diagonal matrix with weights, being the proportion of explained variance by the QTL. The weights for locus $j$ was calculated as $2{p}_{j}(1-{p}_{j}){\alpha }_{j}^{2}$. Note that replacing $\varvec{D}$ by an identity matrix $\varvec{I}$ yields the matrix used for unweighted 2GBLUP.

#### Random forest

Random forest (RF) is an ML model that builds a collection of decision trees, whereby each decision tree is slightly different from the other trees [35]. RF uses the average of all trees using different sets of features to determine the predictions in the test set. The RF model [35] that was used can be written as:

$${\varvec{y}}=\sum_{{t}=1}^{{T}}{{\varvec{h}}}_{{{t}}}\left({\varvec{y}};{{\varvec{W}}}_{{{m}},{{t}}}\right)\boldsymbol{ },$$

in which ${\varvec{y}}$ is the vector of phenotypes, ${{\varvec{h}}}_{{{t}}}\left({\varvec{y}};{{\varvec{W}}}_{m,t}\right)$ is a specific individual regression for tree $t\in (1, T)$, where $T$ is the number of decision trees in the forest, ${\varvec{W}}$ contain the features (SNPs or SNPs combined with QTL), and $m$ is the set of selected features for $t$. The prediction of each tree is obtained by passing down the features in the tree, and the corresponding estimated value at the terminal node is the predicted value. We tuned four hyperparameters, namely n_estimators (number of trees in the forest), max_depth (maximum depth of the tree), min_samples_leaf (minimum number of samples required to be at a leaf node) and max_features (number of features to consider when looking for the best split) (see Additional file 2: Table S1 and Additional file 3), and hyperparameters used in the analysis were shown in Table 1.

**Table 1:** Hyperparameters^1^ used in the machine learning methods

**Method**	**Hyperparameters used**
RF	n_estimators = 1,500, max_features = 2,500
SVR	kernel = rbf

^1^Other parameters that are not mentioned in the table were using default settings (see Additional file 2)

#### Support vector regression

Support vector regression (SVR) uses a linear or nonlinear kernel function to map the input space (the SNP and QTL genotypes in our case) to a higher dimensional feature space, and performs modelling and prediction on the feature space. We tested multiple kernels and tuned two hyperparameters, kernel coefficient gamma and regularization parameter *C* (see Additional file 2: Table S2 and Additional file 4), and decided to use the radial basis function (rbf), and *C* = 1 (default) in our analysis (Table 1).

### Scenarios

QTL were sorted in descending order based on the amount of variance they explained, which was calculated as *2pqa*^*2*^, where *a* represents the simulated additive genetic effect of the QTL. In the first scenario (QTL0), only SNPs and no QTL were included in the model which means that (weighted) 2GBLUP reduces to GBLUP. In the other scenarios, an increasing number of QTL were included in the data. For those scenarios, we use “QTL” followed by a value to refer to a scenario in which the QTL together explained a percentage of variance equal to that value. For example, QTL80 means that the included QTL together explained 80% of the genetic variance. We included scenarios QTL5, QTL10, QTL15, QTL20, QTL40, QTL60, QTL70, QTL80, QTL85, QTL90, QTL95, QTL99 and QTL100. For GBLUP, RF and SVR, all QTL and SNP were given an equal weight. However, for 2GBLUP, all QTL were given an equal weight, while all SNPs were given another equal weight. In weighted 2GBLUP, relative weights (i.e., QTL variance) were provided as well.

For each scenario and each model, accuracy and dispersion bias of predictions were evaluated in the test set. Accuracy was computed as the correlation coefficient between GEBV and TBV, and dispersion bias was computed as the regression coefficient of TBV on GEBV.

### Computational implementation

For GBLUP and 2GBLUP, calc_grm [36] was used to calculate the genomic relationship matrices. Thereafter, MTG2 [37] was used to estimate variance components and GEBV for GBLUP, 2GBLUP and w2GBLUP. For the machine learning models, the Scikit-learn package [38] was used.

## Results

**Fig. 3:**
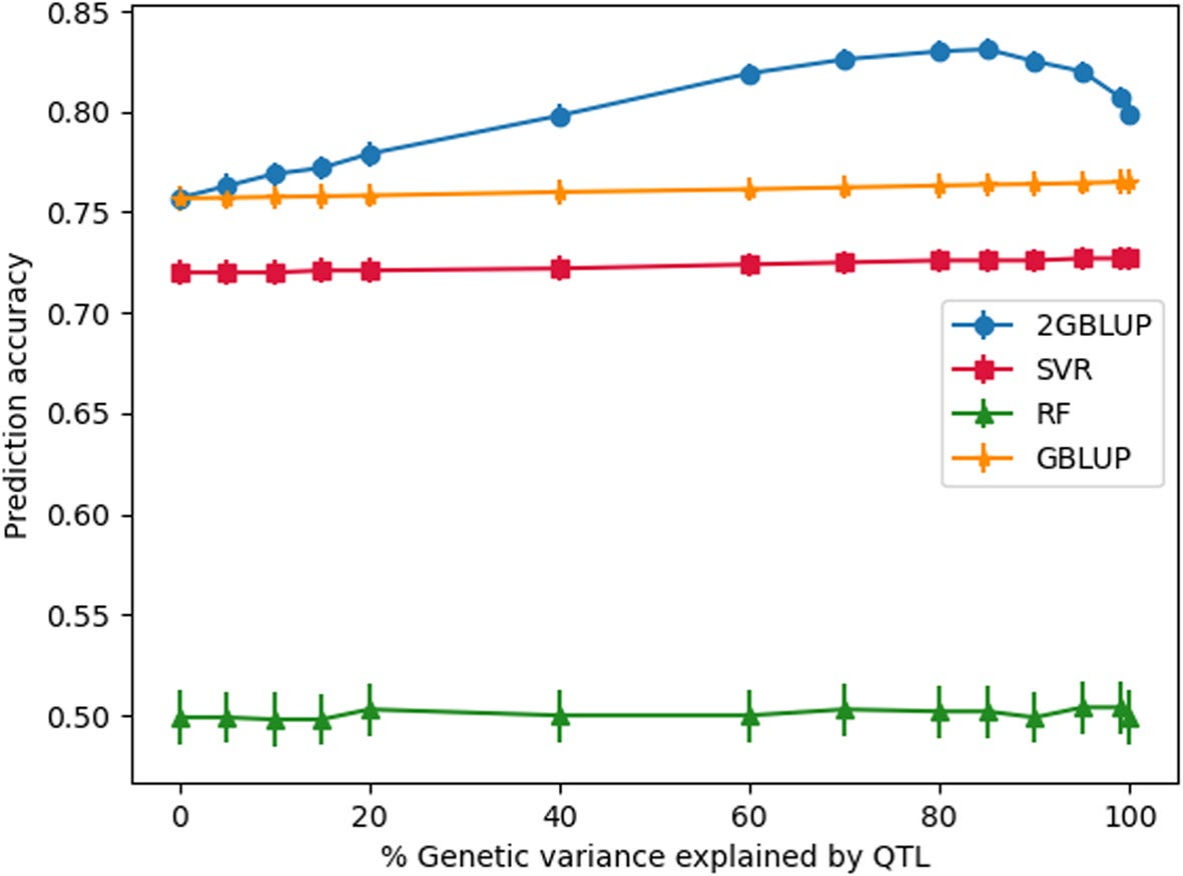
The accuracy of genomic prediction in generation 16 using different genomic prediction methods for different amounts of genetic variance explained by QTL

When there was no QTL included (i.e., QTL0), the prediction accuracy was 0.76 for GBLUP (2GBLUP), 0.72 for SVR, and 0.50 for RF. For 2GBLUP, the accuracy gradually increased when more QTL were included, and peaked at about 0.83 when the included QTL explained around 80% of the genetic variance (Fig. 3). When even more QTL were included, the accuracy dropped and reached a value of 0.80 when all QTL were included. However, at this point, the accuracies were still higher than for GBLUP (0.80 versus 0.76), which indicated that 2GBLUP obtained a higher accuracy than GBLUP for all the scenarios which included QTL. Generally, the accuracy of GBLUP was about 0.04 higher than SVR across scenarios. When more QTL were included in the data, the prediction accuracy of SVR gradually increased, although this increase was very small. Most importantly, the RF model resulted in a much lower accuracy (0.49–0.50) compared to the other models, and the prediction accuracy did not improve by including QTL in the data.

**Fig. 4:**
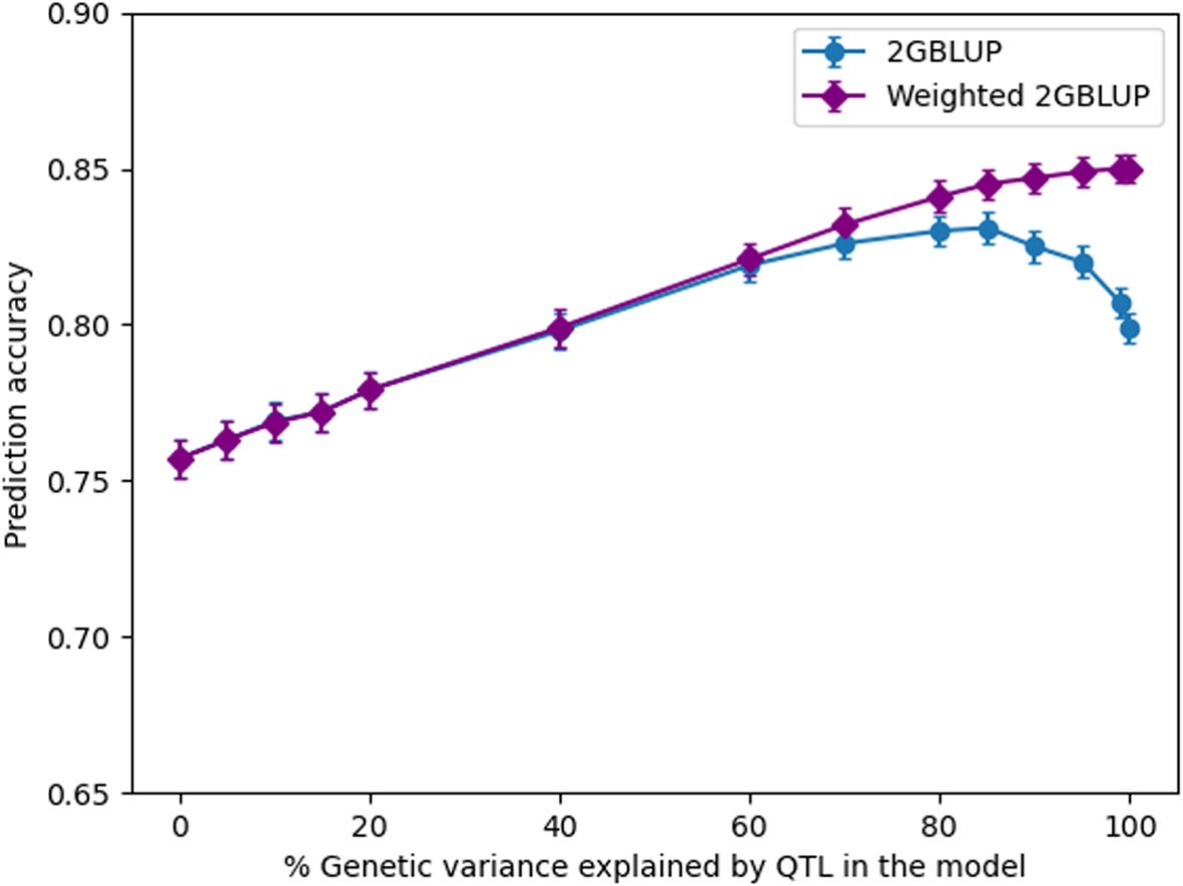
The accuracy of genomic prediction in generation 16 using 2GBLUP and weighted 2GBLUP for different amounts of genetic variance explained by QTL

By weighting QTL in 2GBLUP (w2GBLUP), the prediction accuracy always increased when more QTL were included (Fig. 4). For the scenarios with QTL explaining up to 60% of the genetic variance, the accuracy patterns of 2GBLUP and w2GBLUP were very similar. From QTL60 to QTL100, w2GBLUP had a higher prediction accuracy than 2GBLUP, and the difference in accuracy between w2GBLUP and 2GBLUP gradually increased.

**Fig. 5:**
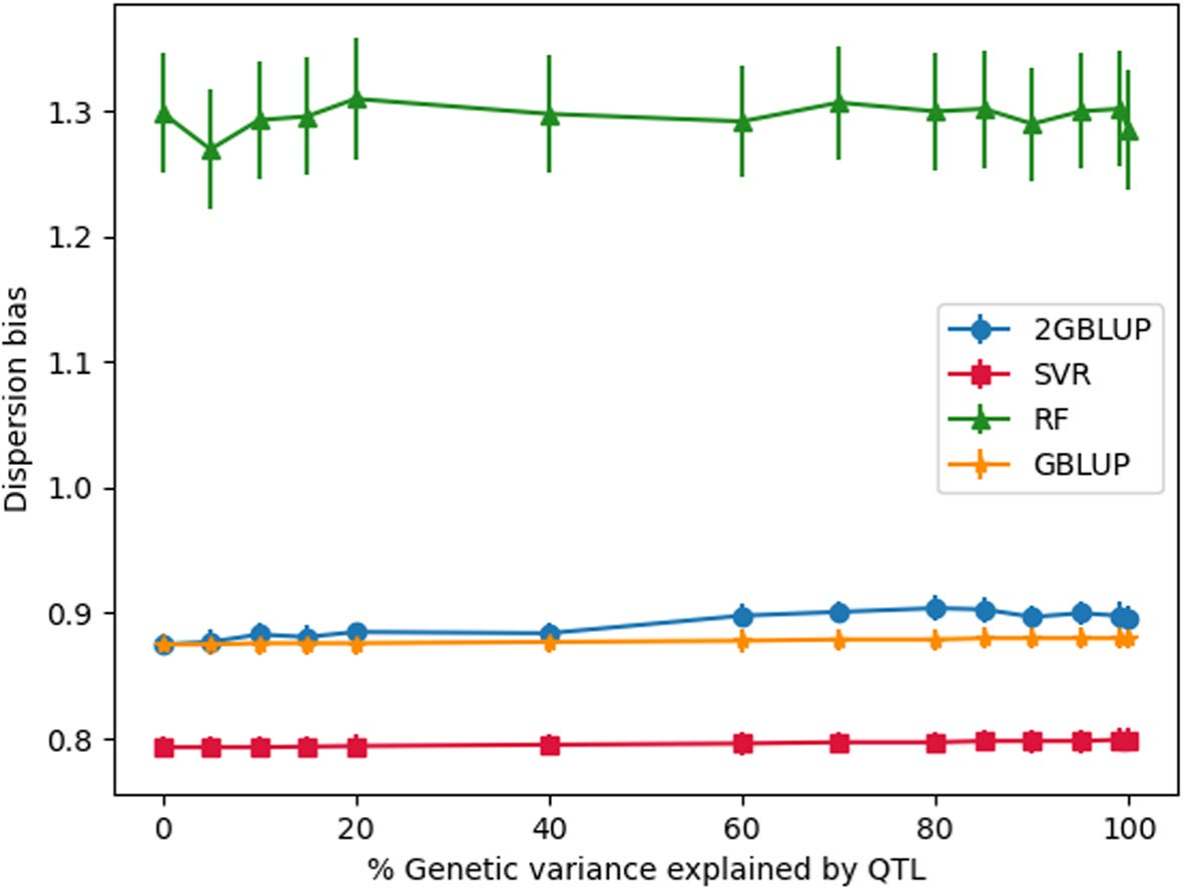
The dispersion bias of genomic prediction in generation 16 using different genomic prediction methods for different amounts of genetic variance explained by QTL

**Fig. 6:**
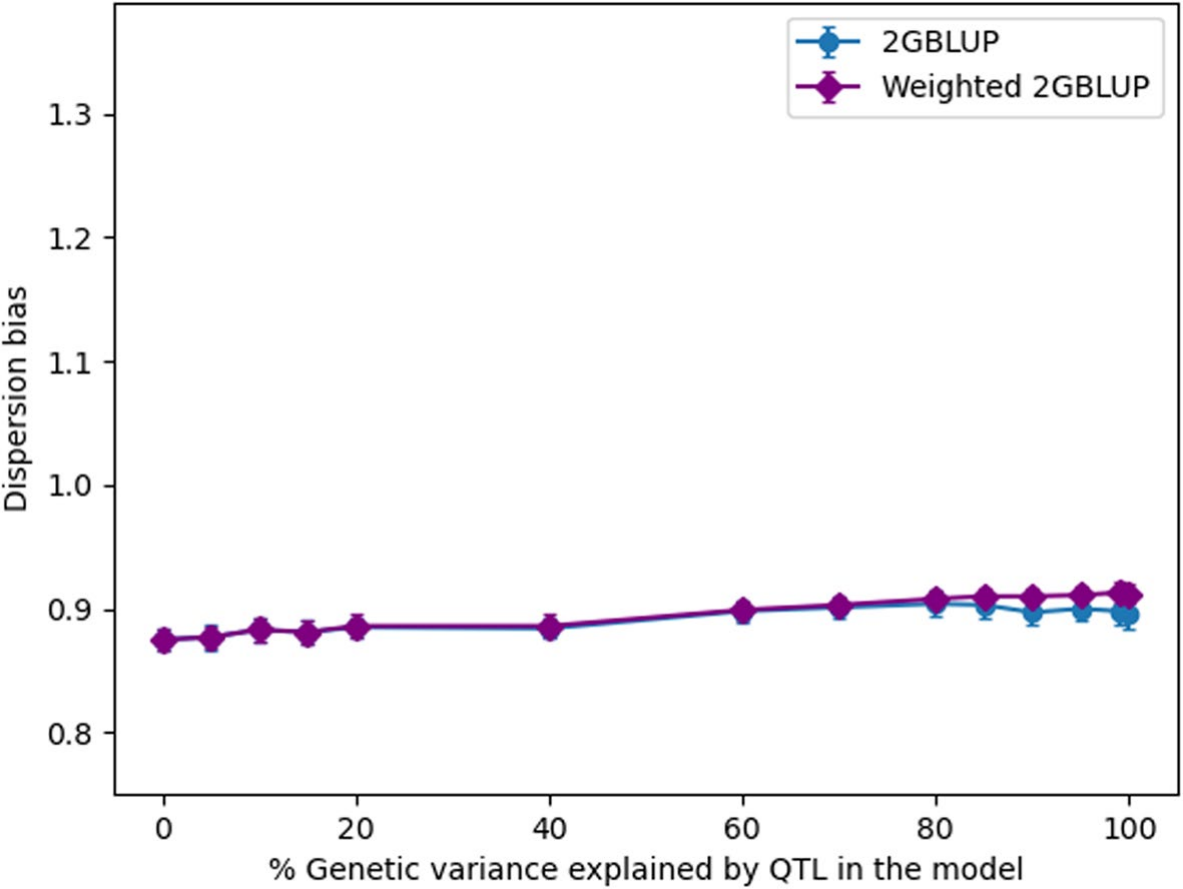
The dispersion bias of genomic prediction in generation 16 using 2GBLUP and weighted 2GBLUP for different amounts of genetic variance explained by QTL

There were nearly no differences in dispersion bias across QTL scenarios, but there were large differences in bias between models (Figs. 5 and 6). The dispersion bias of GBLUP and (w)2GBLUP were similar (~0.90), while the bias was stronger for SVR (~0.80), and RF (~1.29). While no model yielded unbiased GEBV, GBLUP and 2GBLUP had the least dispersion bias.

## Discussion

We aimed to assess the benefit of incorporating QTL information in GBLUP and machine learning models for genomic prediction accuracy, and to find the proportion of genetic variance explained by the QTL included in the model at which the highest prediction accuracy is achieved. Aiming to improve accuracy, we also introduced 2GBLUP, which is a model that separates previously identified QTL from SNP genotypes by including two random genomic effects. Although knowing all QTL is currently optimistic and even unrealistic, it is expected that an increasing number of QTL will be detected in the future. We found that the prediction accuracy of GBLUP, 2GBLUP and SVR can be improved by incorporating QTL genotypes in the dataset. However, the accuracy of GBLUP and SVR increased by less than 0.01 when the included QTL explained all genetic variance, while the accuracy of 2GBLUP increased by 0.07 when the included QTL explained 80% of the genetic variance and increased by 0.04 when the included QTL explained all genetic variance. The prediction accuracy of RF was much lower than other models, and was unaffected by inclusion of QTL genotypes.

It is important to note that the results of this study are based on phenotypes that were simulated from additive (linear) genetic effects only. We chose this approach because breeding values are by definition linearly related to genotypes. If dominance and epistatic effects were present, the results might have been different, potentially favouring the flexibility of ML models. Evaluating the performance of ML models predicting breeding values or phenotypes with non-additive genetic effects will be the focus of our future study.

### Prediction accuracy in 2GBLUP

In this simulation study, we knew all the QTL genotypes and their effects, which made it possible to use 2GBLUP. In practice, however, we do not know the QTL, so an alternative approach is required. This approach can start with GWAS which identifies SNPs that are in high LD with the QTL and will therefore be significantly associated to the trait of interest, i.e., top-SNPs. These top-SNPs can be used in a (weighted) 2GBLUP model in a separate variance component to increase prediction accuracy [28]. For weighted 2GBLUP, the weights can be calculated using the allele frequencies and the estimated SNP-effects, which can be obtained from GWAS directly, or by methods that result in an estimate of the SNP effects such as SNP-BLUP [39] or Bayesian methods [3]. It is also possible to get SNP weights directly; Keel et al. [40] used gene expression information from RNA-seq to obtain SNP weights, and Meuwissen et al. [41] proposed GWABLUP, in which SNP weights can be derived based on the GWAS results.

Our results showed that the prediction accuracy of GBLUP and 2GBLUP increased when QTL information was included, which was in line with results of earlier research. For example, Brøndum et al. [25] identified 1,623 QTL from imputed sequence data using GWAS, and their results showed that including the QTL genotypes improved prediction accuracy, especially when using the 2GBLUP model. Similarly, Raymond et al. [28] included 133 top-SNPs that were identified by a large meta-GWAS. These top-SNPs explained about 10% of the phenotypic variance, and were included in a separate variance component for multi-breed genomic prediction. Their results showed that GBLUP with two genetic effects (where one effect was based on top-SNPs) performed better than GBLUP with a single genetic effect. Altogether, we conclude that including QTL or top-SNPs as a separate genetic effect in the GBLUP model can result in a higher prediction accuracy compared to when QTL/top-SNPs and SNPs are combined in a single effect.

In practice, we expect using only the top-SNPs detected by GWAS in a 2GBLUP model would still lead to higher prediction accuracy compared to regular GBLUP. However, using top-SNPs genotypes will result in a lower accuracy than using QTL genotypes, because the top-SNPs do not fully capture the underlying QTL effects due to imperfect LD.

One of our objectives was to find the optimal proportion of genetic variance explained by QTL to be included in the model to reach the highest accuracy. For GBLUP and SVR, the highest accuracy was achieved when all QTL were included. While for 2GBLUP, the highest accuracy was achieved when the included QTL accumulatively explained 80% of the variance. The prediction accuracy of 2GBLUP decreased when including additional QTL. This decrease may be caused by the assumption of the model that the effects of all variants that are used to build the relationship matrix have a common variance [2, 5]. However, this assumption was severely violated because nearly 25% of the bottom-ranked QTL jointly explained only 1% of the genetic variance. When more small effect QTL were included, the large effect QTL got more shrinkage, which impaired the prediction accuracy. To overcome this, we tested a 2GBLUP model where QTL variances were used as weights to build the QTL relationship matrix (w2GBLUP). When the QTL cumulatively explained more than 80% of the genetic variance, the accuracies of w2GBLUP further increased (Fig. 4). We therefore conclude that using a weighted G matrix can avoid a decrease of accuracy when including progressively more QTL that explained a smaller part of the genetic variance.

It should be noted that the proportion of genetic variance explained by QTL that results in the highest prediction accuracy may depend on the distribution of QTL effect, number of QTL, selection/replacement design and other factors. When these factors change, the optimal fraction of QTL to include in the model may no longer be at 80% explained genetic variance for 2GBLUP. Furthermore, prediction accuracy can be affected by the heritability and reference population size. The impact of these factors on the accuracy deserves a proper investigation and this will be studied in our next study.

### Reasons for low prediction accuracy in RF

The prediction accuracy of RF was about 0.5 for all scenarios, while other models obtained an accuracy above 0.7. This is in contrast to other studies based on real datasets where RF had a comparable prediction accuracy and mean squared error to GBLUP for phenotype prediction [16, 17, 32]. It should also be noted that these studies used the correlation between the predicted values and phenotypes as accuracy, while we used the correlation between the predicted values and true breeding values. However, in our study, the correlation between the predicted values and the phenotype of RF was also significantly lower than for the other models (results not shown).

There are two main reasons for the low accuracy of RF. The first possible reason is that the data structure in this study does not allow RF to fully realize its potential. Machine learning models may perform better than conventional methods when non-additive effects are present, because of their flexibility to deal with complex interactions. In this study, however, we only simulated additive genetic effects, which may limit the benefit of RF. The results of Wang et al. [16] indeed showed that RF had a higher prediction accuracy than GBLUP for litter size in pigs, which is a trait believed to be controlled by substantial dominance and epistasis effects [42].

The second possible reason for the low accuracy of RF is that RF is not designed well for this particular prediction problem. Here we provide three different perspectives to illustrate this. First, RF may not be able to handle a large number of features, which are the SNP and QTL genotypes in our case. Other studies have reported that the performance of RF tends to decline when the number of features becomes very large [43, 44]. However, it is not quite clear whether it is the total number of features, or the proportion of features that are informative that determines the accuracy of RF. We therefore also tested the influence of number of features (SNPs and QTL), and the ratio of informative (QTL) over non-informative (SNPs) features on the accuracy (see Additional file 5: Fig. S1). We simulated a dataset with 20 to 8,179 causal variants (QTL) with a heritability of 1. There are three scenarios: (a) QTL only, (b) QTL combined with SNPs, where the number of SNPs was twice the number of QTL, and (c) QTL combined with a fixed number (10,000) of SNPs. The results showed that the accuracy of RF depended on the total number of informative features, and did not depend on the ratio of number of QTL over number of SNPs. Those results suggest that the accuracy of RF depends on the number of informative QTL underlying the trait, which implies that RF is not suitable for predicting complex traits.

Second, RF might be prone to overfitting. We tested for overfitting by looking at the correlation coefficient between phenotype and predicted value in the training dataset for scenario QTL80. The correlation coefficient for 2GBLUP, RF and SVR were 0.661, 0.986 and 0.805, respectively. In theory, the predicted values should only capture the true breeding value and not any variation in the simulated environmental effect, which results in a maximum expected value of this correlation equal to the square root of the heritability, which in our case is 0.5. Given that all models show a higher correlation than 0.5, we conclude that 2GBLUP, RF and SVR all suffered from overfitting. For RF, the overfitting was extreme, because the correlation coefficient was nearly 1. Other scenarios (with different numbers of QTL included) resulted in a similar outcome (results not shown). Normally, overfitting can be overcome by tuning the hyperparameters, but that strategy was not effective in our study (see Additional file 3). Another option to solve overfitting may be to increase the size of training dataset, such that the number of features does not exceed the number of data points. In this study, the number of features (loci) was four times higher as the number of data points (animals), which was much higher than in other studies [45, 46], and may therefore be an important reason for overfitting.

Third, RF generated incorrect feature importance. We got feature importance from RF directly, and the variance of SNPs from backsolving GBLUP. The backsolved SNP variances were scaled, so that their sum was equal to 1. Then we plotted the scaled SNP variance of GBLUP and the feature importance of RF against the scaled true feature variance. The results showed that the feature variance of GBLUP were generally in line with the true variance, while RF overestimated the importance of few features and underestimated the importance of the majority of features (see Additional file 5: Fig. S2). We find it difficult to explain the difference between overfitting and incorrect feature importance, but maybe it helps with an analogy to cars: The car does not drive (bad performance of RF) because the motor is not running (i.e., feature importance is incorrect), but the real cause may be that there is no gas in the tank (overfitting).

It is worth mentioning that the performance of random forest may be improved by combining it with regularization methods or by performing feature selection [47, 48]. However, these strategies are typically dataset-specific and could reduce the generalizability of our study, which is why we were not explored here.

### Prediction accuracy in SVR

Our results showed that the prediction accuracy of SVR was consistently lower than that of GBLUP, which is in line with one empirical study [17], but in contrasts with the findings of several other studies [16, 49, 50]. There may be two possible reasons for this discrepancy. Firstly, the nature of the dataset may restrict the potential of SVR. While the fully additive genetic signals under this data structure match with the assumptions of GBLUP, this is not necessarily the case for SVR, whose potential benefits of capturing non-linear genetic effects cannot be utilized here. In the presence of non-additive genetic effects, SVR may have benefit over GBLUP [16, 49, 50]. Secondly, SVR is prone to overfitting. When many markers (QTL and SNPs) are included, it seems that GBLUP effectively integrates all marker information through the genomic relationship matrix, making it more robust to noise. Whereas SVR may not distinguish signal from noise efficiently.

### Trend in accuracy over generations

It is well known that the accuracy of genomic prediction decays over generations when reference populations are not updated [3, 51–53], which is caused by less strong relationships between selection candidates and reference animals when they are more generations apart [53–55]. Another explanation for this is that the LD between SNPs and QTL decays over generations [56]. The focus of this study was to include QTL genotypes as features in genomic prediction, which alleviates the necessity of LD between the SNPs and QTL. Therefore, genomic prediction accuracy may persist better across generations when QTL are included. To test this hypothesis, we compared the accuracy of predictions across generations 16 to 20, while still using the original training dataset (generation 11–15) and randomly selecting 500 animals per generation from generations 17 to 20 to add to the test set.

The results showed that when the included QTL explained 5% of genetic variance (scenario QTL5), the rate of decline over generations was similar for GBLUP, 2GBLUP, SVR and RF (see Additional file 6: Fig. S1a). However, when QTL that explained 80% of genetic variance were included (scenario QTL80), the accuracy of 2GBLUP decreased less compared to GBLUP (see Additional file 6: Fig. S1b). Therefore, the drop in accuracy across generations was similar for GBLUP, RF and SVR for the QTL5 and QTL80 scenarios, while for 2GBLUP the drop was lower with QTL80. We found that the persistency in accuracy across generations was not improved by including QTL information in GBLUP, SVR and RF. Depending on the proportion of variance explained by the included QTL, the accuracy of 2GBLUP was more persistent than GBLUP, which indicates that 2GBLUP is able to mitigate to some extent the impact of relationship or LD decay on the prediction accuracy.

## Conclusions

This study investigated the impact of incorporating QTL information into genomic prediction models, comparing GBLUP models with the ML models RF and SVR. Our findings show that incorporating QTL genotypes in GBLUP and SVR can improve prediction accuracy. The extent of improvement varies across models, i.e., 2GBLUP benefits most from incorporating QTL while the incorporation of QTL resulted a limited increase in prediction accuracy of SVR and GBLUP. Moreover, GBLUP consistently resulted in a higher prediction accuracy than SVR across all scenarios. These findings suggest that if more QTL information becomes available, the selection of an appropriate model is crucial for optimizing prediction performance. In contrast to (w)2GBLUP, RF did not show improvements when QTL information was included, and had a much lower accuracy than the other models. Two possible reasons for this result are that the data structure in this study does not allow RF to fully realize its potential, and that RF is not designed well for this particular prediction problem. These results highlight the importance of selecting appropriate models for genomic prediction and underscore the potential limitations of machine learning models when applied to genomic prediction in livestock.

## Supplementary Information

Additional file 1. QMSim input file. This file contains the QMSim input file of simulated data.

Additional file 2. Parameters that machine learning methods required. This file contains the lists of parameters that used in random forest and support vector regression.

Additional file 3. Parameter tuning process of random forest. This file contains the detail of parameter tuning process of random forest.

Additional file 4. Parameter tuning process of support vector regression. This file contains the detail of parameter tuning process of support vector regression.

Additional file 5: Fig. S1. Prediction accuracy of RF and GBLUP using simulated dataset with different number of informative QTL. a) QTL only, b) QTL combined with SNPs that close to the QTL, and the number of SNPs was twice the number of QTL and c) QTL combined with fixed numberof SNPs. Fig. S2. Real QTL variance against variance or importance obtained from a) GBLUP and b) RF.

Additional file 6: Fig. S1. The accuracy of genomic prediction using different genomic prediction methods for a) 5% of genetic variance explained by QTL and b) 80% of genetic variance explained by QTL across generations.

## Data Availability

QMSim input file used to generate the data during this study is Additional file 1. Seeds for QMSim simulation and codes for analysis can be found via https://git.wageningenur.nl/yang110/.

## Abbreviations

GEBV: Genomic estimated breeding values
GP: Genomic prediction
GWAS: Genome-wide association studies
LD: Linkage disequilibrium
ML: Machine learning
QTL: Quantitative trait loci
RF: Random forest
SNP: Single nucleotide polymorphism
SVR: Support vector regression
TBV: True breeding values
w2GBLUP: Weighted 2GBLUP
WGS: Whole genome sequence
