# Supplementary material for: Incorporating information of causal variants in genomic prediction using GBLUP or machine learning models in a simulated livestock population
Source: J Anim Sci Biotechnol. 2025 Aug 19;16:118. doi: 10.1186/s40104-025-01250-5 (PMC12362903; doi:10.1186/s40104-025-01250-5)
Supplement: Supplementary file 1 — Additional file 1. QMSim input file. This file contains the QMSim input file of simulated data. [file 40104_2025_1250_MOESM1_ESM.docx]

Additional file 1: QMSim input file

/*******************************

** Global parameters **

*******************************/

title = "sequence";

nthread = 12;

nrep = 1; //Number of replicates

h2 = 0.25; //Heritability

qtlh2 = 0.25; //QTL heritability

phvar = 1.0; //Phenotypic variance

/*******************************

** Historical population **

*******************************/

begin_hp;

hg_size = 2800 [0] //Size of the historical generations

2000 [2250]

200 [2500]

10000 [2505];

nmlhg = 20; //Number of males in the last generation

end_hp;

/*******************************

** Populations **

*******************************/

begin_pop = "p1";

begin_founder;

male [n = 20, pop = "hp"];

female [n = 5000, pop = "hp"];

end_founder;

ls = 1; //Litter size

pmp = 0.5 /fix; //Proportion of male progeny

ng = 20; //Number of current generations

md = rnd;

dr = 0.5;

sr = 1.0;

sd = ebv /h;

ebv_est=accur 0.80 /true_av;

begin_popoutput;

data / gen 11 12 13 14 15 16 17 18 19 20;

genotype /snp_code /gen 11 12 13 14 15 16 17 18 19 20;

allele_freq /gen 15;

end_popoutput;

end_pop;

/*******************************

** Genome **

*******************************/

begin_genome;

begin_chr = 29;

chrlen = 100; //Chromosome length

nmloci = 2000; //Number of markers

mpos = rnd; //Marker positions

nma = all 2; //Number of marker alleles

maf = eql; //Marker allele frequencies

nqloci = 350; //Number of QTL

qpos = rnd; //QTL positions

nqa = all 2; //Number of QTL alleles

qaf = eql; //QTL allele frequencies

qae = rndg 0.42; //QTL allele effects

end_chr;

mmutr = 1e-4 /recurrent; //Marker mutation rate

qmutr = 1e-5 /recurrent; //QTL mutation rate

r_mpos_g; //Randomize marker positions across genome

r_qpos_g; //Randomize QTL positions across genome

//interference = 25;

select_seg_loci /maft 0.001;

end_genome;

/*******************************

** Output options **

*******************************/

begin_output;

linkage_map;

allele_effect;

hp_stat;

end_output;
