# Supplementary material for: Incorporating information of causal variants in genomic prediction using GBLUP or machine learning models in a simulated livestock population
Source: J Anim Sci Biotechnol. 2025 Aug 19;16:118. doi: 10.1186/s40104-025-01250-5 (PMC12362903; doi:10.1186/s40104-025-01250-5)
Supplement: Supplementary file 2 — Additional file 2. Parameters that machine learning methods required. This file contains the lists of parameters that used in random forest and support vector regression. [file 40104_2025_1250_MOESM2_ESM.docx]

Additional file 2: Parameters that machine learning methods required

**Table S1** Parameters that random forest package required

| **Parameters/Option** | **Description** | **Tuning(Default)/Default** |
| --- | --- | --- |
| n_estimators | The number of trees in the forest | Tuning(Default=None) |
| Criterion | The function to measure the quality of a split | Default=”squared_error” |
| max_depth | The maximum depth of the tree | Tuning(Default=None) |
| min_samples_split | The minimum number of samples required to split an internal node | Default=2 |
| min_samples_leaf | The minimum number of samples required to be at a leaf node | Tuning(Default=1) |
| min_weight_fraction_leaf | The minimum weighted fraction of the sum total of weights (of all the input samples) required to be at a leaf node. | Default=0 |
| max_features | The number of features to consider when looking for the best split | Tuning(Default=None) |
| max_leaf_nodes | Grow trees with max_leaf_nodes in best-first fashion | Default=None |
| min_impurity_decrease | A node will be split if this split induces a decrease of the impurity greater than or equal to this value | Default=0 |
| bootstrap | Whether bootstrap samples are used when building trees | Default=True |

**Table S2** Parameters required by support vector regression

| Parameters/Option | Description | Tuning(Default)/Default |
| --- | --- | --- |
| kernel | Specifies the kernel type to be used in the algorithm. Option: ‘linear’, ‘poly’, ‘rbf’, ‘sigmoid’. | Tuning(Default=‘rbf’) |
| gamma | Kernel coefficient for ‘rbf’, ‘poly’ and ‘sigmoid’. Option: ‘scale’, ‘auto’ or float. | Tuning(Default=‘scale’) |
| tol | Tolerance for stopping criterion. | Default=1e-3 |
| *C* | Regularization parameter. The strength of the regularization is inversely proportional to *C*. | Tuning(Default=1) |
