# Supplementary material for: Incorporating information of causal variants in genomic prediction using GBLUP or machine learning models in a simulated livestock population
Source: J Anim Sci Biotechnol. 2025 Aug 19;16:118. doi: 10.1186/s40104-025-01250-5 (PMC12362903; doi:10.1186/s40104-025-01250-5)
Supplement: Supplementary file 3 — Additional file 3. Parameter tuning process of random forest. This file contains the detail of parameter tuning process of random forest. [file 40104_2025_1250_MOESM3_ESM.docx]

Additional file 3: Parameter tuning process of random forest

We used the python package sklearn.ensemble.RandomForestRegressor [36] for hyperparameter tuning and data analysis. Parameters and/or options are listed (see Additional file 2: Table S1).

We did not tune min_samples_split because it is correlated with min_samples_leaf.

The hyperparameters were tuned based on one extra replicate. The resulting optimized combination of hyperparameters was used for the analyses for all replicates. In the tuning process, only datasets with SNP markers were used, while the correlation of predicted valued and TBV of animals in generation 16 was used as prediction accuracy.

First, we tuned the parameter n_estimators. Generally, more trees in the forest makes the results more reliable, but it requires more computational resources. We tried values for n_estimators of 50, 125, 250, 500, 1,500, 3,000, 7,000, 10,000, and 15,000, while using default settings for other parameters (Fig. S1). When n_estimators was 7000, the computation time was 7 days, therefore we did not consider values higher than 3000. The accuracy when using values for n_estimators of 50 and 125 were 0.266 and 0.378, respectively, which were lower than that of others (> 0.43). So we did not use 50 and 125 as n_estimators in our further analysis.


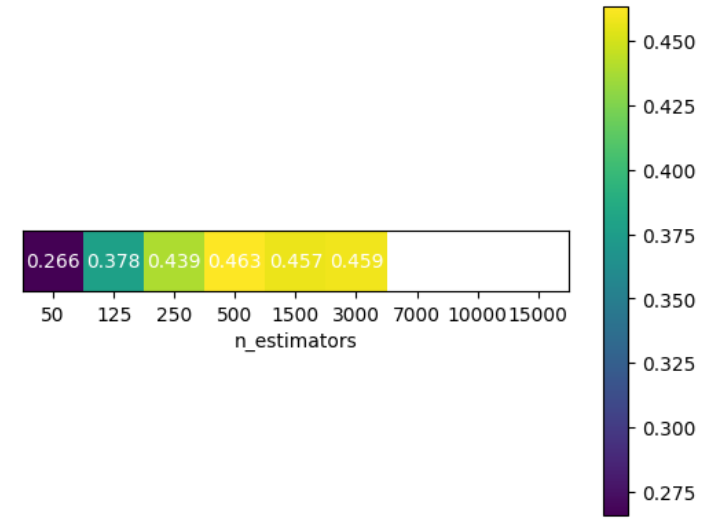


**Figure S1** Prediction accuracy for different values of n_estimators while using default settings for other parameters

Second, we fixed n_estimators at 250 to tune max_depth. We considered values of 5, 50, 100, 1,000, 2,500, 5,000, 10,000, 20,000, 40,000 and no limits. The results showed that only the scenario with max_depth of 5 produced a minimum accuracy of 0.343, while all other scenarios have an accuracy of 0.439 (Fig. S2). This indicates that when the depth of the tree is greater than a certain value (50 for this dataset), there is no effect on accuracy. Therefore, we decided to use the default setting of no limit.


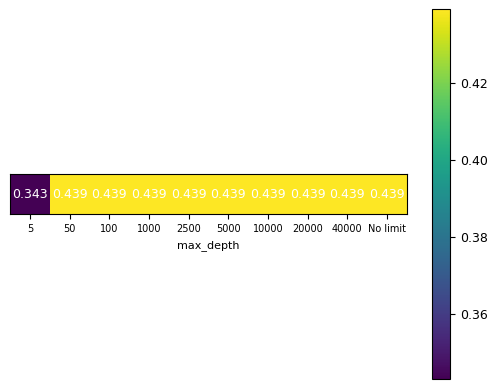


**Figure S2** Prediction accuracy for different values of max_depth when n_estimators was 250 and using default setting for other parameters

Thirdly, for each value of n_estimators, 250, 500, 1,500, 3,000, 7,000, 10,000, 15,000, we combined it with max_feature of 500, 1,000, 2,500, 5,000, 10,000, 20,000, considering the potential interaction between n_estimators and max_features (Fig. S3). Here we also included the value of n_estimators larger than 7,000, because computation time decreased with a restriction of max_feature. For min_leaf, the default setting of 1 was used. We found that all the groups achieved the highest accuracies with max_features of 2,500, except for the group with n_estimators of 250 where the highest accuracy was achieved with no limit for the feature number. Therefore, we decided to choose 2,500 as max_features setting in our further analysis. Furthermore, the computation time was more than 15 hours when n_estimators was larger than 7,000, while accuracy was only slightly (~0.05 with max_features=2,500) higher. Therefore, we didn’t include n_estimators values larger than 7,000 in our further study, and fixed max_features to 2,500.


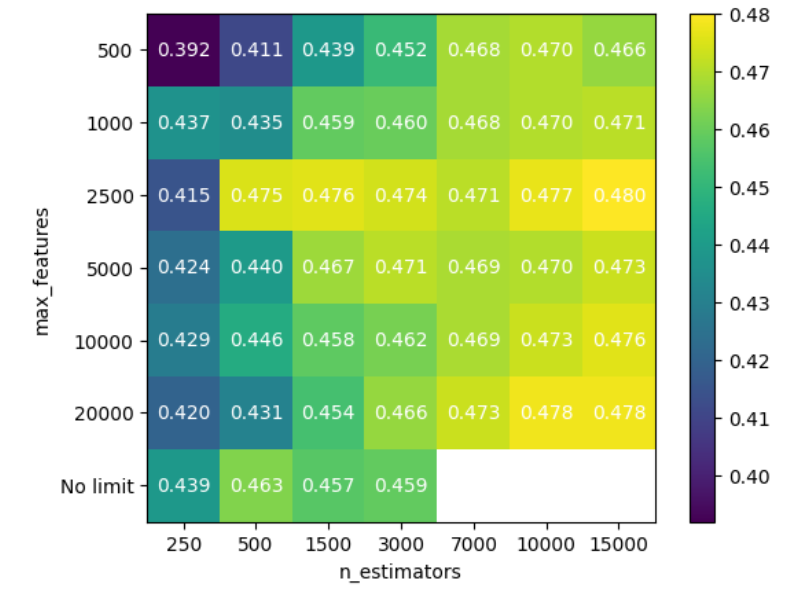


**Figure S3** Prediction accuracy for different combinations of n_estimators and max_features, while using default setting for other parameters

Finally, we tuned min_leaf. We considered values of 3, 10, 20, 100, 500, 2,500, 5,000, 10,000, 15,000 as min_leaf when using 250 as n_estimators (Fig. S4). We found that when min_leaf increased from 1 to 20, prediction accuracy slightly increased. When min_leaf increased further from 20 to 15000, accuracy gradually decreased to 0. Thus, we only used 3, 10, 20 as min_leaf to test the interaction with n_estimators (Fig. S5). The results show that other n_estimators had the same accuracy pattern as 250. The accuracy dipped from min_leaf of 1 to 3, while increased from 3 to 20, showing the highest accuracy with min_leaf=1. Based on these results, we used the default setting (1) for min_leaf in further analysis.


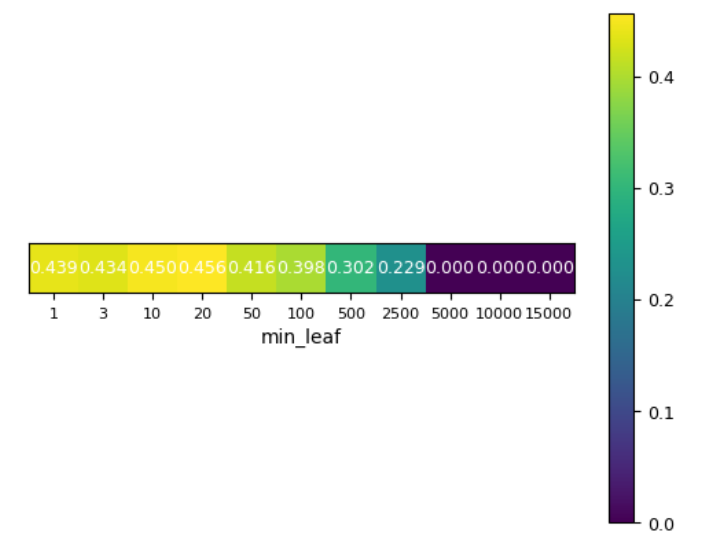


**Figure S4** Prediction accuracy for different values of min_leaf when n_estimators was 250 and using default setting for other parameters


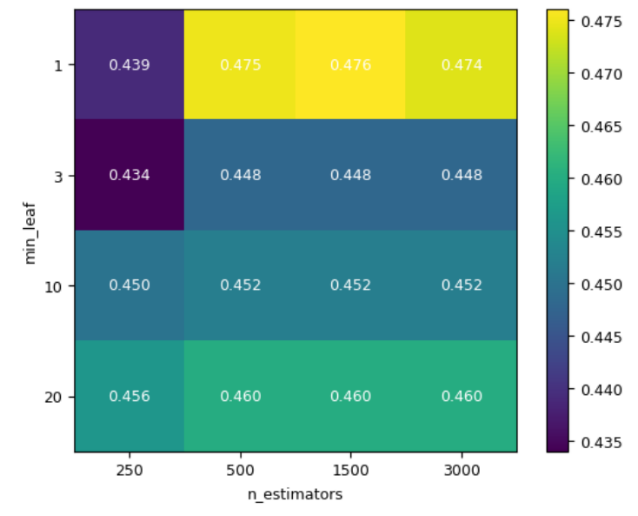


**Figure S5** Prediction accuracy for different combinations of n_estimators and min_leaf combinations when max_features was 2,500 and using default setting for other parameters

Considering the balance between accuracy and computation cost, we decided to use n_estimators of 1,500.
