# Supplementary material for: Incorporating information of causal variants in genomic prediction using GBLUP or machine learning models in a simulated livestock population
Source: J Anim Sci Biotechnol. 2025 Aug 19;16:118. doi: 10.1186/s40104-025-01250-5 (PMC12362903; doi:10.1186/s40104-025-01250-5)
Supplement: Supplementary file 4 — Additional file 4. Parameter tuning process of support vector regression. This file contains the detail of parameter tuning process of support vector regression. [file 40104_2025_1250_MOESM4_ESM.docx]

Additional file 4: Parameter tuning process of support vector regression

We used the python package sklearn.svm.SVR [36] for hyperparameter tuning and data analysis. Parameters and/or options are listed (see Additional file 2: Table S2).


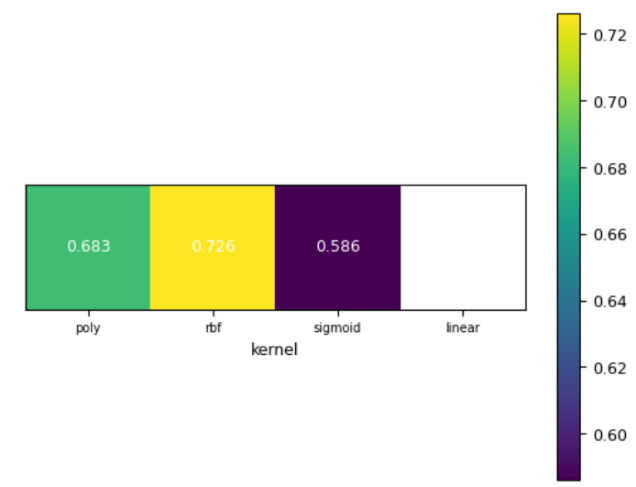
We started the tuning process by choosing a kernel. We used the same simulated data as was used in the tuning process of RF and again used the correlation between predicted values and TBV of animals from generation 16 as accuracy. We considered the kernels ‘poly’, ‘rbf’, ‘sigmoid’, and ‘linear’, respectively, while using default settings for other parameters.

**Figure S1** Prediction accuracy for different kernel in SVR while using default setting for other parameters

We found that kernel ‘rbf’ reached the highest accuracy, so we used ‘rbf’ in our further tuning (Fig. S1). Then we tuned gamma. Both the ‘auto’ and ‘scale’ option are related to the number of features which is not fixed in our dataset, so we did not consider using a fixed number. We tried the options ‘auto’ and ‘scale’. We found that ‘scale’ resulted in a higher accuracy than ‘auto’, so we used the default setting, ‘scale’, in the further tuning process (Fig. S2).


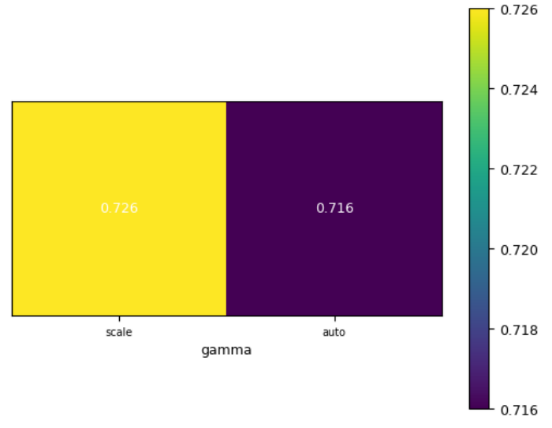


**Figure S2** Prediction accuracy for different gamma options in SVR while using default setting for other parameters

Finally, we tuned parameter C, using 0.001, 0.01, 0.1, 0.4, 1, 4, 7, 10, 100, 1,000. We found that the prediction accuracy reached the highest accuracy when C was 1 (Fig. S3).


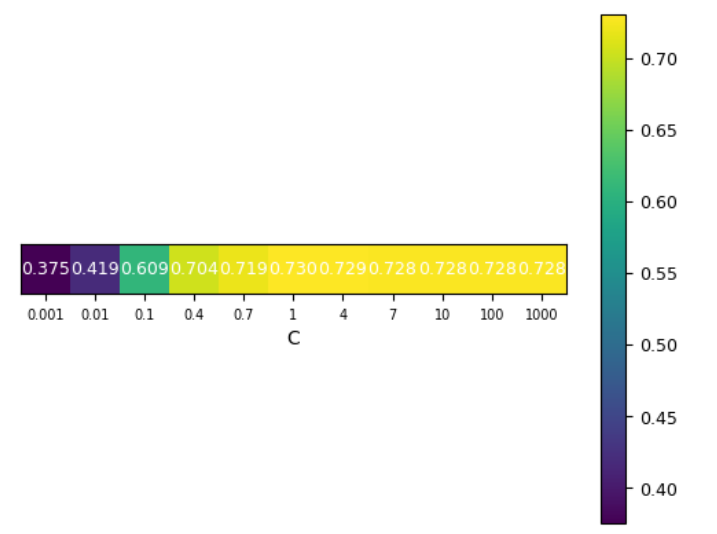


**Figure S3** Prediction accuracy for different values for C in SVR while using default setting for other parameters

So we decided to use C of 1.
