# Supplementary material for: Incorporating information of causal variants in genomic prediction using GBLUP or machine learning models in a simulated livestock population
Source: J Anim Sci Biotechnol. 2025 Aug 19;16:118. doi: 10.1186/s40104-025-01250-5 (PMC12362903; doi:10.1186/s40104-025-01250-5)
Supplement: Supplementary file 5 — Additional file 5: Fig. S1. Prediction accuracy of RF and GBLUP using simulated dataset with different number of informative QTL. a) QTL only, b) QTL combined with SNPs that close to the QTL, and the number of SNPs was twice the number of QTL and c) QTL combined with fixed numberof SNPs. Fig. S2. Real QTL variance against variance or importance obtained from a) GBLUP and b) RF. [file 40104_2025_1250_MOESM5_ESM.docx]

Additional file 5


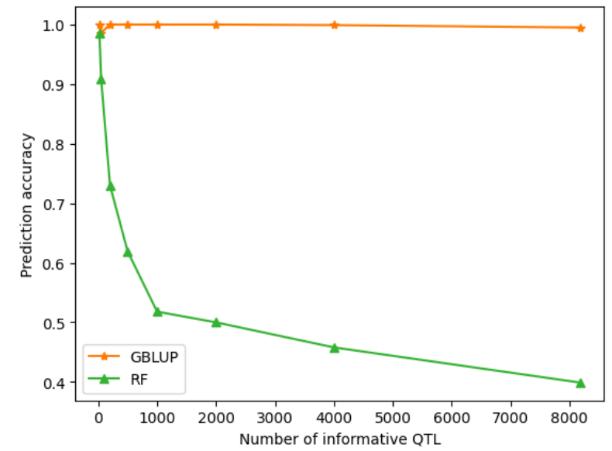


a


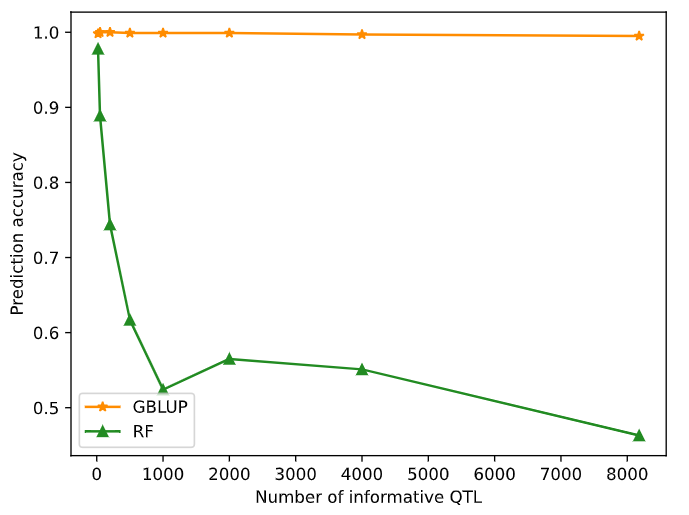


b


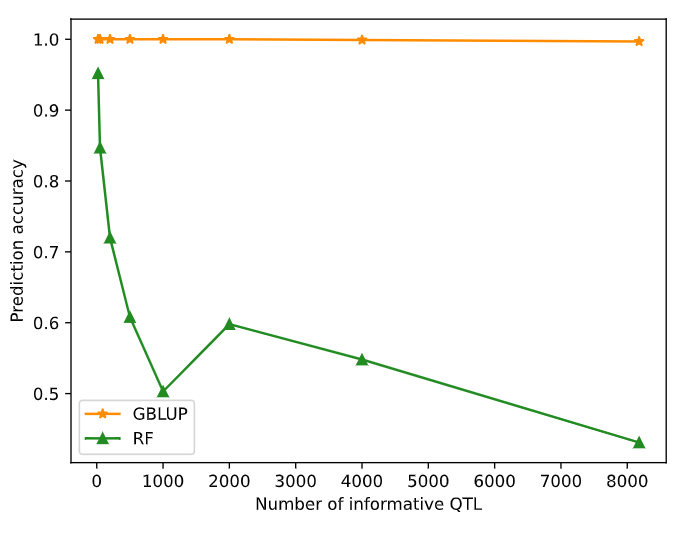


c

**Figure S1** Prediction accuracy of RF and GBLUP using simulated dataset with different number of informative QTL. a) QTL only, b) QTL combined with SNPs that close to the QTL, and the number of SNPs was twice the number of QTL and c) QTL combined with fixed number (10,000) of SNPs


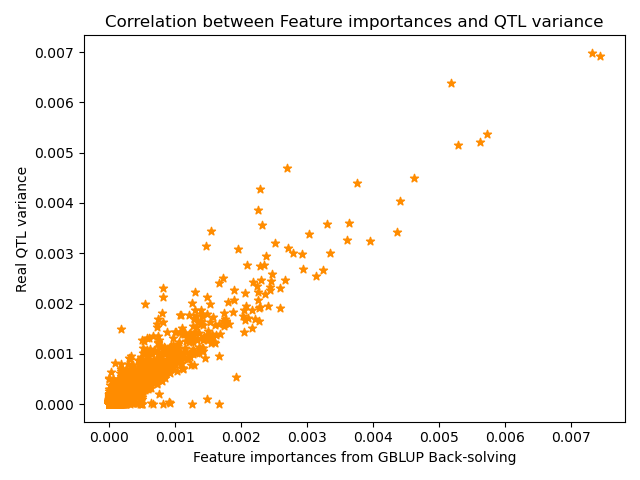

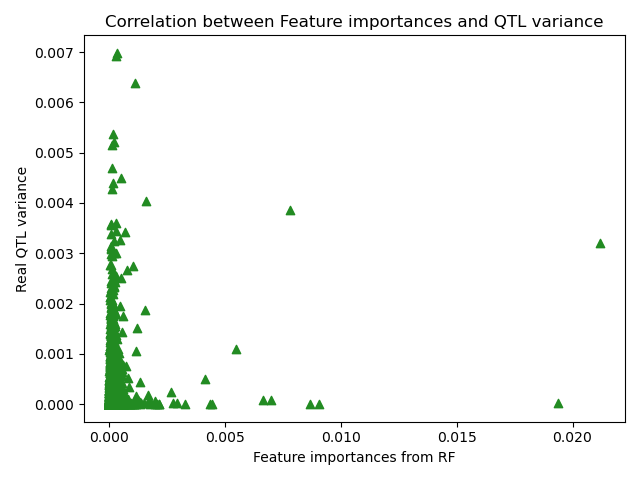


a b

**Figure S2** Real QTL variance against variance or importance obtained from a) GBLUP and b) RF
