# Supplementary material for: Incorporating information of causal variants in genomic prediction using GBLUP or machine learning models in a simulated livestock population
Source: J Anim Sci Biotechnol. 2025 Aug 19;16:118. doi: 10.1186/s40104-025-01250-5 (PMC12362903; doi:10.1186/s40104-025-01250-5)
Supplement: Supplementary file 6 — Additional file 6: Fig. S1. The accuracy of genomic prediction using different genomic prediction methods for a) 5% of genetic variance explained by QTL and b) 80% of genetic variance explained by QTL across generations. [file 40104_2025_1250_MOESM6_ESM.docx]

Additional file 6


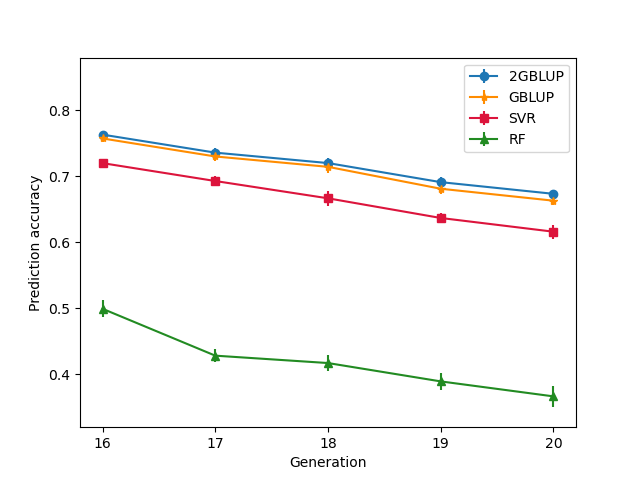

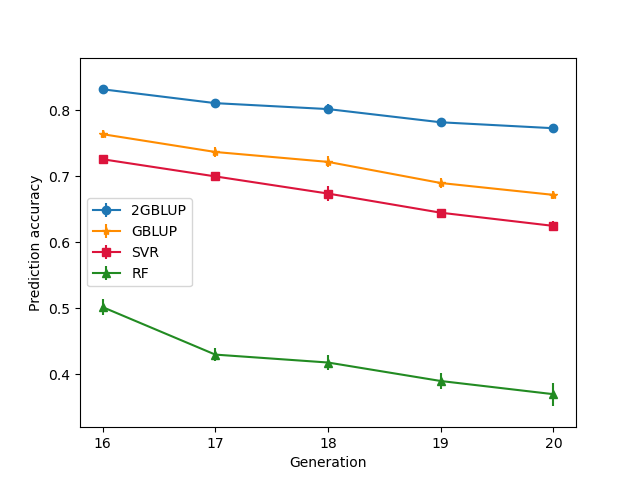


a b

**Figure S1** The accuracy of genomic prediction using different genomic prediction methods for a) 5% of genetic variance explained by QTL and b) 80% of genetic variance explained by QTL across generations.
